# Positioning, power, and identity transformation among English teachers in Chinese private universities: a narrative inquiry

**DOI:** 10.3389/fpsyg.2025.1729332

**Published:** 2026-01-12

**Authors:** Qi Jin, Jungyin Kim

**Affiliations:** Department of English Education, Jeonbuk National University, Jeonju, Republic of Korea

**Keywords:** Chinese private universities, emotional strain, narrative inquiry, positioning theory, teacher identity, willingness capability power framework

## Abstract

**Introduction:**

Although language teacher identity has been widely examined in public university contexts, relatively little is known about how teachers in resource-constrained private institutions navigate role ambiguity and emotional strain. This study aims to investigate how English teachers at a private university construct and transform their teacher identities over time within structural and discursive constraints.

**Methods:**

This study utilized qualitative methods and a narrative inquiry across a multiple case study framework. Data were collected from three English teachers through semi-structured interviews, classroom and meeting observations, and netnographic methods. This study traced the pre-positioning, negotiated positioning, and performed positioning of identity construction through positioning theory, which explores how individuals assume professional roles through words and actions. The willingness, capability, power (WCP) framework was used to analyze how teacher identity transformation was influenced by willingness to act, skills to act, and power to act.

**Results/findings:**

The findings from RQ1 identified three themes. In pre-positioning, all three participants’ entry into the teaching profession was accidental and reflected gendered discourses. Negotiated positioning manifested as role conflict, emotional costs, and adaptive reframing. Within the performed positioning, teachers stabilized their identities through specialization and strategic boundary-setting. RQ2 identified four WCP-based identity trajectories: Unfulfilled Initiative, where strong motivation was hindered by limited power; Adaptive Reframing, which involved adjusting expectations and teaching methods to maintain engagement; The Residual Functionality reflected the emotional withdrawal amid disempowerment; and Reinforcing Engagement, which manifested as the alignment of motivation, capability, and recognition to support sustained professional growth.

**Discussion:**

This study provides a power-focused and temporally grounded account of identity transformation in the context of resource-constrained private institutions. The research demonstrates that identity is a dynamic and emotionally mediated process. Two context-specific concepts, discursively orphaned identity and performed stagnation, highlight subtle forms of disempowerment that are often overlooked in formal policy discourse. The findings suggest that institutional support should go beyond material resources to encompass discursive inclusion and symbolic affirmation. This provides theoretical insights for future research and policymaking in the field of language teacher education in resource-constrained private higher education settings.

## 1 Introduction

Teacher identity refers to the sense of self that teachers continually construct in relation to their professional roles and the contexts of their work, which is central to the development and the choices teachers make regarding their instruction (Beijaard et al., 2004; Van Lankveld et al., 2017). In recent years, studies about teacher identities concentrating on language teachers have become a burgeoning focus within language teacher education (e.g., Chen et al., 2024; Mercer and Kostoulas, 2018; Morton, 2018). Early research on language teacher identity have focused primarily on understanding its dynamics (Tsui, 2007), multidimensionality (Farrell, 2011), and emotional tension (Barkhuizen, 2016; Song, 2016). This series of studies effectively explored the micro, meso, and macro-level factors influencing identity construction (De Costa and Norton, 2017). Recent research (e.g., Bao and Feng, 2022, 2023; Bowen et al., 2021; Yang and Yin, 2024) highlights that university language teachers negotiate their identities within complex institutional environments. Unlike primary and secondary school teachers, university teachers need to balance competing demands related to teaching, research, and service (Van Lankveld et al., 2017). These complexities are intensified by unique institutional pressures, such as academic evaluation pressures (Tran et al., 2017) and research expectations (Barkhuizen, 2021).

In China, however, most research on the identity of university language teachers focuses on public universities (e.g., Bao and Feng, 2023; Ren and Pan, 2025; Yang et al., 2022). The main research themes revolve around the balance between teaching and research (Yang et al., 2022) and professional negotiation (Ren and Pan, 2025). The narratives and experiences of English teachers working in private universities in China are still lacking (Chen, 2024; Zhang, 2025). The increasing demand for teaching since the early 21st century has led to a rapid expansion of higher education, resulting in a diverse landscape of educational institutions (Zhang, 2025). This has given rise to a dual-track higher education system, with public and private sectors operating in parallel with different funding sources and employment arrangements (Lin et al., 2005). Public universities, funded by the state and offering *bianzhi*-based employment, are seen as the mainstream (Si, 2023). *Bianzhi* in Chinese is often referred to as the “iron rice bowl,” which is the metaphor that is a symbol of the job security, stable income, and social prestige (Si, 2023). In contrast, private universities rely on tuition fees and face regulatory and financial precarity (Zhang, 2025). Teachers in private universities experience low social recognition, receiving lower salaries, limited benefits, and heavier teaching loads compared to their public university counterparts (Lin et al., 2005). Research-oriented faculty are often scarce due to a teaching-centric culture and weak internal research incentive mechanisms (Li and Morgan, 2008). Teachers frequently resign due to the insufficient salary, low level of job satisfaction and lack of professional recognition (Liu, 2020). These structural disadvantages collectively influence faculty perceptions of their own value, role negotiation, and their institutional and professional positioning (Zhang, 2025).

Unlike STEM disciplines such as engineering or mathematics, which tend to involve more technical and standardized forms of knowledge, English language teaching involves particularly complex identity dynamics as English teachers often assume the roles of a language instructor, a cultural intermediary, and emotionally invested professional (Pennington and Richards, 2016). Roles that are further exacerbated in private universities where students are observed to have lower proficiency and motivation (Liu, 2020). This is largely because students applying to private universities generally score lower in the national college entrance examination compared to students applying to public universities (in the lowest 25%), as private universities have relatively lower entrance requirements and less competition (Zhang, 2025).

Although existing research (e.g., Song, 2016; Trent, 2011; Yang and Han, 2022) has examined how personal and contextual factors influence teacher identity, less attention has been paid to the mechanisms underlying positional shifts; that is, how and why individuals move from one identity position to another (Kayi-Aydar, 2015). Harré and Van Langenhove (1991) proposed positioning theory, which conceptualizes identity as interactionally and discursively constructed, and socially and institutionally contextualized (Harré et al., 2009). The willingness, capability, power (WCP) framework extends positioning theory by considering how personal desires, abilities, and contextual power structures influence the identity positions individuals can assume (Davies and Harré, 1999).

Recent studies have combined these two frameworks (e.g., Hardiana et al., 2025; Huang and Wang, 2024). However, they typically treat these as separate analytic tools and focused on pre-service teachers situated in structured, high-support environments. This study advances the literature by using positioning theory and WCP as a dynamic explanatory mechanism, revealing not only the positions teachers occupy but why they accept, resist, or reframe them at particular moments (Van Langenhove et al., 2016). Rather than treating WCP as a supplementary lens, this study theorizes identity transformation as a process that is simultaneously shaped by discursive positioning and fluctuations in WCP. This study also extends the application of the combination of positioning theory and WCP to a new context: in-service English teachers working in Chinese private universities in China. This context is characterized by resource scarcity, role ambiguity, and institutional asymmetry (Zhang, 2025). Methodologically, this study employs the narrative inquiry method in a multi-case study design to analyze the identity trajectories of three in-service English teachers.

## 2 Literature review

### 2.1 Teacher identity

Recent scholarship has moved beyond essentialist views of teacher identity as fixed and role-bound, instead embracing more dynamic and context-sensitive perspectives (Barkhuizen, 2016). The sociocultural perspective argues that identity is formed through participation in collective activities (Johnson, 2009), while the poststructuralist approach emphasizes its construction process at the discursive and relational levels (Arvaja, 2016; Norton, 2013).

Within English language teaching (ELT), identity research shares these fundamental assumptions while introducing specific disciplinary concerns (Kayi-Aydar, 2019). Initial research (Kumaravadivelu, 2003; Pavlenko, 2003) focused on identity and language, especially how non-native English speaking teachers (NNESTs) negotiated with legitimacy and linguistic capital vis-a-vis native-speaker expectations. Later research, informed by sociocultural theory, examined how English teacher identity is shaped through institutional participation and engagement in professional communities (Johnson, 2009; Tsui, 2007). Poststructuralist studies further highlighted English teacher identity as intersectional, emerging at the nexus of language, race, gender, and class, and discursively constructed through positioning (De Costa and Norton, 2017; Kayi-Aydar, 2015). Teachers in ELT contexts are required to combine linguistic knowledge with contextual sensitivity and a high level of reflection (Pennington and Richards, 2016). In the current research, this study examines EFL teacher identity within poststructuralist frameworks which emphasize the role of discourse, institutional arrangements, and personal narratives as key shaping elements over time and across contexts (Mockler, 2011).

### 2.2 Positioning theory

Aligning with poststructuralist views of identity, positioning theory situates the construction of identity in practices of self and other positioning through discourse in narrative forms that seek to rationalize lived experience (Davies and Harré, 1999). These narratives provide access to positions that enable the construction, negotiation, and re-configuration of identities in interaction (Harré and van Langenhove, 1991).

To account for the temporal development of identity, Harré and Dedaić (2012) proposed three stages: pre-positioning (imagined roles shaped by personal history and prior experiences), negotiating positioning (roles adjusted through interactions with institutional actors), and performing positioning (formed identities that are relatively stable in practice but still adaptable to the environment).

Davies and Harré (1999) suggested the WCP framework to explain the conditions under which positioning succeeds or fails. Willingness defines the internal motivation of an individual to adapt to the specific identity role. Capability includes all the emotions, thoughts, and the physical-level resources required to play those roles. Power operates at two levels. At an interpersonal level, it is through the granting or denial of legitimacy in the interaction. At an institutional level, it is through the structures and resources that constrain or promote identity roles (Harré and Moghaddam, 2003). Huang and Wang (2024) proposed that, even in the presence of motivation and competence, the absence of power is a significant barrier to positioning.

Early formulations of positioning theory tended to treat discursive and structural dimensions as emerging simultaneously in local interactions (Harré and Van Langenhove, 1991). However, scholars have since pointed out that such accounts may underplay the broader cultural dimensions of identity (Linehan and McCarthy, 2000). To address this issue, this study draws on Foucault (Foucault, 1970, 1980) to conceptualize power as both embodied in discourse and embedded in institutional systems that shape and regulate identity positioning.

### 2.3 The application of positioning theory in teacher identity research

Positioning theory has started to gain interest in education, with early applications centering on spoken discourse (e.g., Davies and Harré, 1999). Deppermann (2013) extended positioning theory into narrative contexts. Building on this narrative turn, Kayi-Aydar (2021) emphasizes the necessity of exploring positioning theory to examine how identities unfold in the context of time, space and institutions.

Empirical research has largely followed two strands: one centers on personal histories, the other on institutional discourse and discursive power. For example, Kayi-Aydar (2018) found that three Latina pre-service teachers used emotionally charged experiences to connect with Latino students and distance themselves from those from different cultural backgrounds. In contrast, Skog and Andersson (2015) documented how Swedish pre-service mathematics teachers were positioned as passive and disempowered within dominant institutional and linguistic discourses, reinforcing asymmetries in discursive power relations.

More recent studies have begun to engage both agency (willingness and capability) and power, though these elements are still often examined separately. In this study, agency refers to the motivational, cognitive, attitudinal, and behavioural resources teachers draw on to regulate and promote learning (Heikonen et al., 2017; Pietarinen et al., 2016; Toom et al., 2015). Willingness captures only the motivational and attitudinal aspects of agency, while capability reflects its cognitive and skill-based elements (Davies and Harré, 1999; Harré and Moghaddam, 2003). Agency shapes how teachers interpret and enact their roles, it forms an important basis for identity development (Ruohotie-Lyhty and Moate, 2016; Wu, 2022). Weng (2023) explored how a novice Chinese EAP teacher in the U.S. enacted agency to legitimize his professional role, and yet institutional constraints such as mandated curricula were treated as fixed and not as co-constructed. Sun et al. (2022) offered a valuable relational lens, and their discussion of contextual affordances (e.g., training programs) opens up further possibilities for examining how such supports may be dialogically negotiated or resisted in situated practice.

Parallel to these international developments, against the ongoing education reforms, research in China has increasingly examined how teachers, at both K-12 and post-secondary levels, construct, sustain, and enhance their identity over the past decade (e.g., Jiang and Zhang, 2021; Lee et al., 2013; Trent and Liu, 2023). Yet positioning theory has been used selectively to examine how institutional structures and discursive practices shape identity (Shi, 2022; Wu, 2022). Systems characterised by collectivist values, hierarchical management, and performance-driven evaluation (Zhang, 2025) place teachers under considerable emotional and role-based strain, making positioning theory particularly valuable for analysing how identity roles are assigned, legitimised, resisted, or transformed (Huang and Wang, 2024).

Existing Chinese studies provide insightful but partial accounts. Huang and Wang (2024) applied the WCP framework to analyze three identity trajectories, acceptance, concession, and persistence, among student teachers in Hong Kong, but approached WCP as an outcome typology rather than a dynamic process. Shi (2022) described discursive resistance among the staff of a bilingual school without a longitudinal scope. Xing et al. (2025) scrutinized identity work during a Lesson Study and incorporated emotional dimensions, focusing on a single case. In Chinese higher education, the application of positioning theory remains relatively underexplored. Wu (2022) traced the transformational journey of an EFL teacher, demonstrating how pursuing a doctoral degree enabled her to move from marginalization to empowerment. However, the micro-level discursive positioning moments embedded in her everyday teaching were not the main analytic concern.

Taken together, both international and Chinese studies have made significant contributions to our understanding of teacher identity. However, opportunities remain to further theorize how willingness, capability, power interact in shaping identity transformation, particularly among in-service teachers. In addition, more longitudinal and narrative-oriented research is needed to examine identity transformation as an evolving and situated process, especially in the underexplored setting of private universities. Therefore, the current work uses positioning theory alongside the WCP framework to articulate English teacher identity as a fluid, complex, and layered construct in Chinese private universities.

### 2.4 The current study

The study is centered around the following research questions:

1. How do three English teachers at one Chinese private university perceive their identities as reflected in their constructed narratives across different career stages (i.e., pre-positioning, negotiated positioning and performed positioning)?
2. What roles do willingness, capability, power (WCP) play in shaping the transformation of three English teachers’ identities within this private university setting?

**Figure 1:**
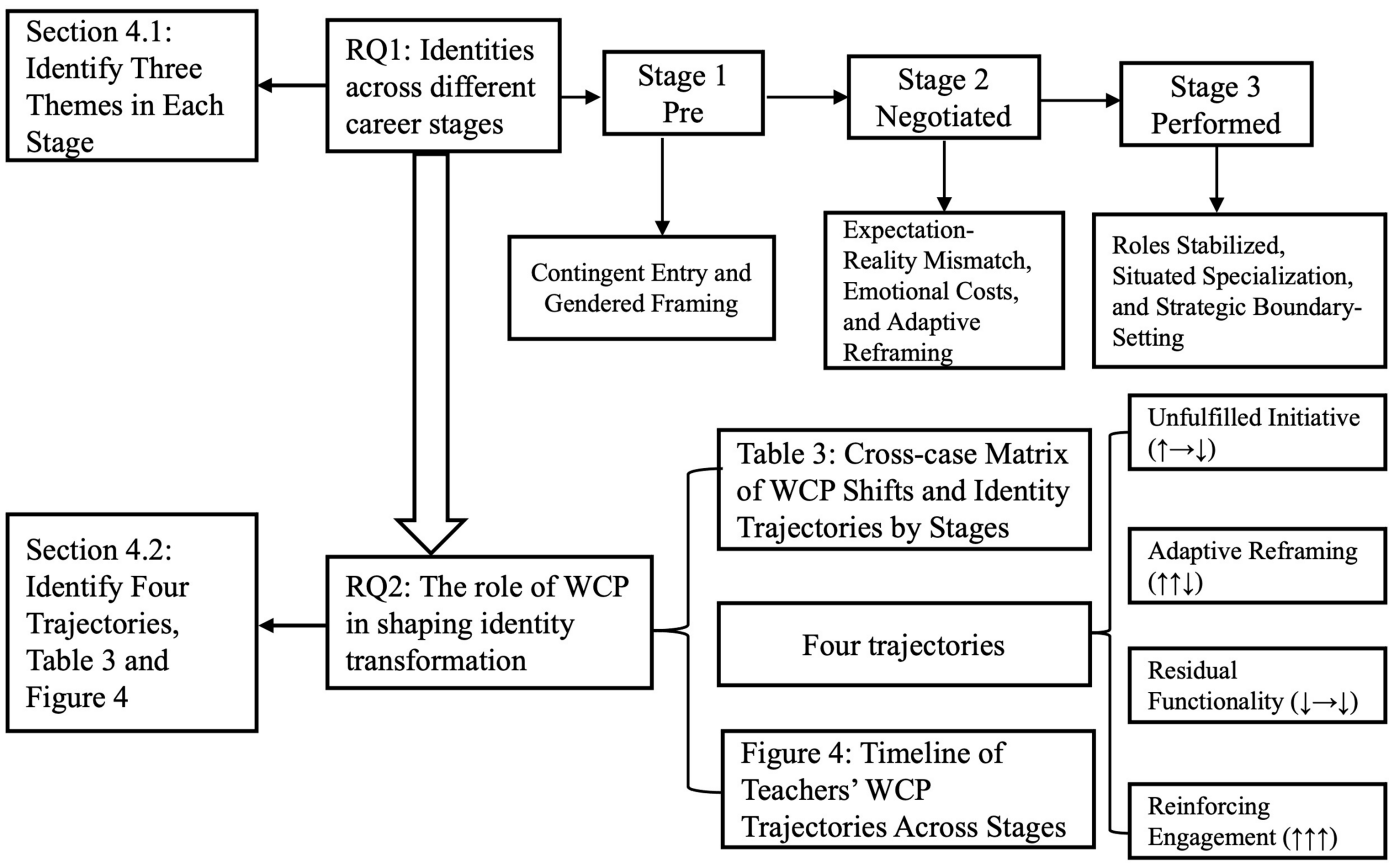
Mapping of research questions to findings.

Figure 1 shows how the two research questions were addressed and matched with the findings.

## 3 Methodology

To obtain an in-depth understanding of how and why teacher positioning changes through WCP, this study adopted a qualitative multiple case study design integrated with narrative inquiry. Case studies involve in-depth analysis of a single unit (e.g., an individual, group, or event), emphasizing dynamic factors relevant to context (Feagin et al., 2016). As Yin (2017) points out, case studies enable researchers to draw rich details from multiple data sources, allowing for in-depth description and explanation of complex phenomena. This triangulation approach aligns with best practices in narrative research, ensuring a comprehensive understanding of participants’ identity trajectories (Barkhuizen, 2016; Clandinin and Connelly, 2000).

Narrative inquiry considers participants’ stories as reconstructions of the participants’ interpretations of their past and present experiences which suits tracing identity movements across the career stages and institutional context (Tsui, 2007). Narrative inquiry by considering the temporal and relational aspects of the experiences enriched the case descriptions and highlighted the intricacies of identity negotiation (Li, 2025; Sonday et al., 2020).

### 3.1 Research context

As the researchers were collecting their data between December 2024–June 2025, the university was undergoing “the Private University Qualification Assessment,” a national accreditation process initiated by the Ministry of Education. This assessment aimed to evaluate whether the private institutions have met the state required institutional governance, faculty qualifications, instructional resources, and student outcomes. Those that successfully passed the assessment were confirmed a university legitimacy, and had qualification to offer officially recognized degree programs. In the English department, this resulted in more scrutiny to instructional materials, more frequent changes to the syllabi, and more pressure on students to evaluate the course.

The institution’s records indicated that a large number of the teachers in the Business English Faculty had master’s degrees, and the institution’s policies was to have them encouraged to obtain a doctorate degree.

### 3.2 Participants selection and background

A combination of purposive, convenience, theoretical, and maximum variation sampling was employed to recruit three English teachers from a private university in southeastern China.

The institutional site was purposively selected based on three criteria: (1) a mid-sized organizational structure with accessible faculty; (2) administrative openness to research; and (3) ongoing engagement in the Private University Qualification Assessment, which heightened discursive pressures relevant to identity negotiation. Among several candidates, one university was selected for its alignment with these criteria. Access to this institution was convenience-based (Etikan et al., 2016) as the researcher had a personal acquaintance in another department unrelated to English language teaching. Through this contact, the researcher was introduced to the Foreign Languages Department, where several teachers voluntarily expressed interest in participating. The individual served only to facilitate initial contact and was not involved in recruitment or data collection.

Within this institution, purposive sampling was used to recruit information-rich participants whose experiences aligned with the study’s aim (Coyne, 1997). Participants were full-time English teachers of the Business English major in the Foreign Languages Department of this Chinese private university. Business-related English teaching often involves applied, interdisciplinary content and heightened role expectations, which intensify identity negotiation and positioning. Recruiting from the same department minimized contextual variability related to curriculum, policy, and evaluation. Eligible participants had a minimum of 1 year of working experience to ensure initial exposure to institutional structures. They also participated based on their voluntariness to reflect on and articulate their professional journeys. Researchers also consulted the university website for Business English faculty profiles and reached out via email to explain their initial research plan. Finally, seven teachers responded positively to the recruitment, representing the majority of Business English faculty in this small department. While efforts were made to include male participants, gender imbalance is common in Chinese higher education (Li and Horta, 2024), where female teachers comprise over 80% of English language faculty (Zhang et al., 2024). Only one male teacher initially expressed interest but later withdrew. Additionally, another teacher declined participation due to discomfort with classroom observation, reducing the available pool to five candidates.

Then, theoretical sampling was employed, involving sampling decisions based on analytic ground developed during the research process (Bagnasco et al., 2014). Guided by positioning theory and WCP framework, the researchers sought participants who could illuminate distinct identity positioning trajectories and WCP dynamics across career stages. Simultaneously, to ensure diversity in age, educational background, and professional experience, researchers employed maximum variation sampling to encompass a wide range of perspectives and life events (Patton, 2002).

The remaining five candidates were spoken to informally. Two novice (1–3 years) teachers were ultimately selected, since early career stage tends to have more identity tension (Vanegas et al., 2021). Despite being in the same career stage, T1 and T2 were in different age brackets, and had different degrees and prior experiences. T1, who had doctoral qualifications and considerable experience teaching in high school, was almost 40 years. T2, on the other hand, was in her mid 20s, had only just completed her master’s, and had no teaching experience. Such contrasting profiles gave variation in the ways early career teachers manage the demands of the system. In addition, T1 was more research active while T2 was more in the early stage of her career where research was not a focus. A third participant (T3), a teacher with over 15 years of experience, was selected to offer a more seasoned perspective and contribute retrospective insights into earlier identity negotiations. Two additional candidates were excluded for theoretical reasons. One mid-career teacher’s trajectory closely mirrored that of T1, offering little additional analytic variation. One experienced teacher was excluded due to marked reluctance to engage with sensitive but central topics such as institutional governance, emotional strain, and identity conflict. Her abstract and cautious responses lacked the depth, personal meaning-making, and storied qualities required in narrative inquiry (Clandinin and Connelly, 2000).

This study did not pursue statistical universality, but rather aimed for theoretical representativeness by intentionally incorporating diversity within and between different career stages, thereby achieving analytical universality through cross-case comparisons and theoretical abstraction (Ayres et al., 2003). Despite diverse backgrounds, the identity transformations all converged around a common trajectory driven by WCP, reflecting a pattern that transcends individual cases (Polit and Beck, 2010). This convergence and diversity enhanced the theoretical relevance of the study and its transferability to similar private university environments, aligning with the goal of analytic generalization (Firestone, 1993).

Finally, theoretical saturation was used as the criterion for determining sample adequacy. Theoretical saturation refers to the point in data collection where no new themes or conceptual insights emerge, and the properties and interrelationships of key categories are fully developed (Aldiabat and Le Navenec, 2018; Hennink and Kaiser, 2022). By the time interviews with the three selected participants were completed, no additional themes related to identity positioning or WCP dynamics had emerged. Cross-case comparisons further elaborated the interconnections among categories, indicating that saturation had been achieved (Saunders et al., 2018). As noted earlier, additional candidates failed to provide new theoretical variation or deepen the emergent categories. A summary of the participant profiles is provided in Table 1.

**Table 1:** Participant background information.

Participant	Education	Prior experience	Joined	Current focus	Gender	Age
T1	BA & MA in Econ/Finance (abroad); PhD in Education (China, 2023)	Corporate; international high school	2023	Research-oriented (70% research)	Female	Late 30s
T2	BA in English (China); MA in International Education (UK)	Brief corporate experience	2023	Teaching-focused (low research involvement)	Female	Mid 20s
T3	Dual BA in English & Trade; MA in Management	Public university; international trade	2009	Practitioner (over publishing)	Female	Early 40s

### 3.3 Data collection

Semi-structured interviews were primary data collection methods, while netnography and subsequent observational methods were employed for triangulation. Two or three informal conversations of 20 to 30 min each were carried out but not analyzed to build rapport and refine interview prompts. Each participant completed a semi-structured interview that lasted 60–90 min, either in person or on Tencent Meeting, at their convenience. Using the WCP model and positioning theory, the interview protocol employed non-directive questions to enable participants to tell their stories as freely as possible (Adams, 2015). The Critical Incident Technique (CIT, Butterfield et al., 2005) served to take the participants to major events and/or turning points in their professional histories. Every interview for this study was conducted in the Chinese language but translated and subsequently member-checked for accuracy (Birt et al., 2016).

The netnographic database consists of (1) public documents (e.g., institutional web pages, WeChat Moments, chat documents); (2) elicited documents (six different teaching materials, six drafts of a thesis with supervisor feedback, and three private reflective writings); and (3) the researcher’s field notes documenting online positioning activities (Kozinets, 2010). Reflexivity remained by analytic memos and reflexive journaling as well as by not employing evaluative language. Given the constraints of the participants’ schedules, two classroom observations (each 45 to 60 min) and one meeting observation were done as time permitted. Observational field notes recorded the fluidity of identity, the complexities of role negotiation, and dynamics of institutions.

### 3.4 Data analysis

**Figure 2:**
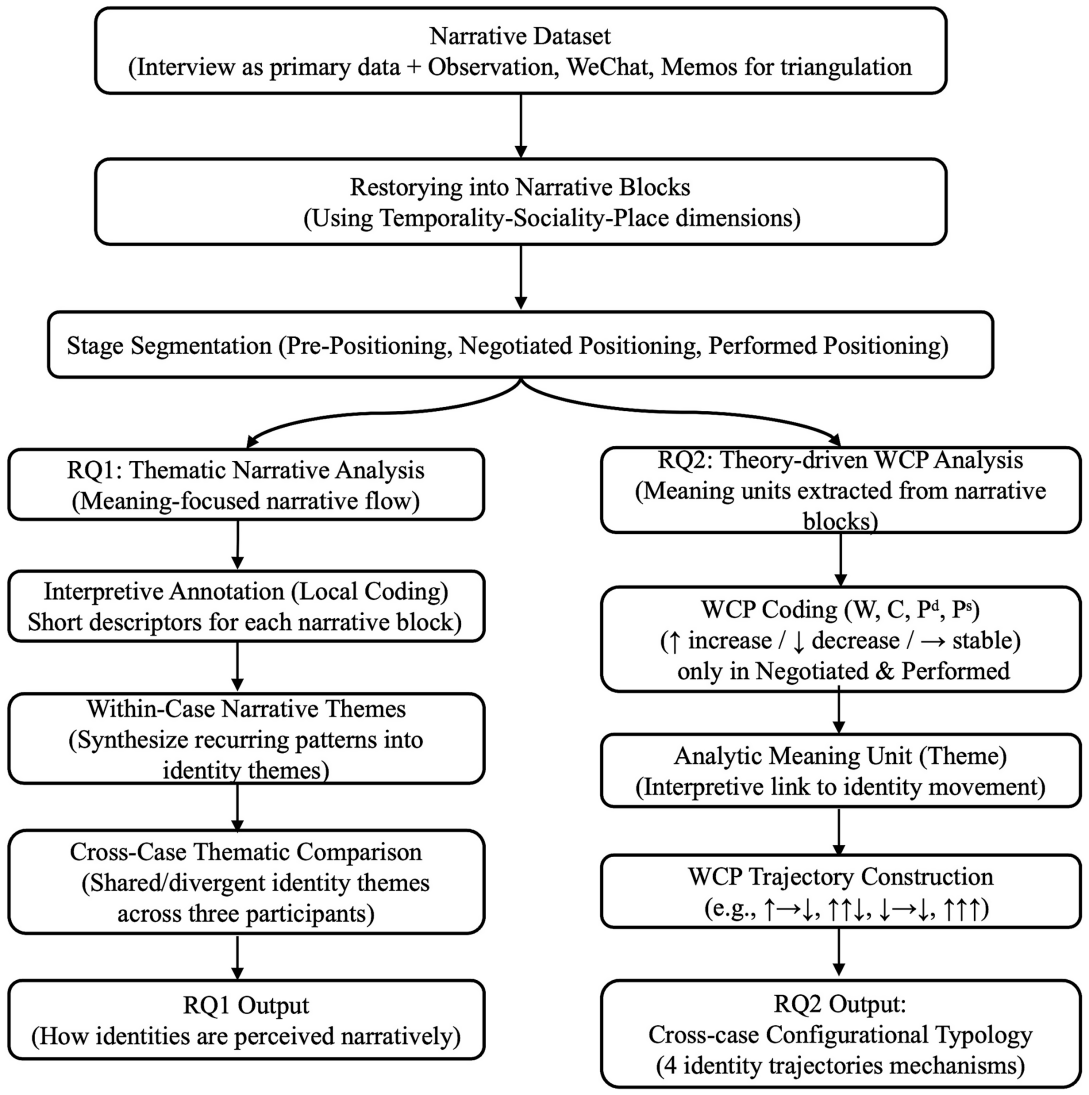
Coding and analysis flowchart for RQ1 and RQ2.

This study employed a two-pronged analytical strategy to address research questions. RQ1 adopted a stage-based thematic narrative analysis to examine how teacher identities evolved across time, roles, and relationships. RQ2 applied a theory-driven thematic analysis combined with configurational analysis to theorize the mechanisms of identity transformation through the WCP framework. While both analyses drew on the same dataset, their analytic logics and units of analysis diverged. RQ1 focused on narrative blocks as the unit of analysis, preserving story flow while identifying recurrent themes (Riessman, 2008). For RQ2, the analytic unit shifted to WCP meaning units, abstracted interpretive segments extracted from narrative blocks. While the data remained narrative in origin, the analytic focus shifted from the narrated to the explained, from story to structure. These units were synthesized into directional WCP trajectories (e.g., ↑ → ↓) to theorize how patterned shifts across dimensions generated distinct identity transformation mechanisms. This analytic separation allowed the study to examine identity as both a narrated process (RQ1) and a mechanism-driven transformation (RQ2). A visual summary of the overall coding process is provided in Figure 2.

#### 3.4.1 Thematic narrative analysis for RQ1

The data analysis followed a general thematic approach, categorized as “narrative analysis” (Barkhuizen et al., 2014). This analysis is similar to thematic analysis in that it involves finding common themes in existing narrative data and focuses more on what is said than on how it is said (Riessman, 2008).

All interviews were manually transcribed by the first author to ensure deep familiarization. Then, we repeatedly reviewed the original Chinese interview materials and shaped them into coherent narrative blocks. This process was informed by Clandinin and Connelly’s (2000) three-dimensional framework (temporality, sociality, and place), which helped trace how experiences unfold over time, are embedded in relationships, and are shaped by institutional and physical contexts. These narrative blocks were not arbitrary coding units but experiential events with internal coherence, often centered around identity-related turning points (Craig, 2007). Semi-structured interviews served as the primary data source. Supplementary materials, including classroom and meeting observations, WeChat posts, and analytic memos, were not coded separately but used to triangulate, verify, and contextualize participants’ narratives (Wu et al., 2025).

Each participant’ s narrative was segmented into three positioning stages, pre-positioning, negotiated positioning, and performed positioning, and manually coded in Excel as Stage 1, 2, and 3. Segments were annotated with interpretive descriptors (e.g., emotional distancing, role ambiguity), then grouped by stage to generate stage-based themes (e.g., emotional costs in Stage 2, situated specialization in Stage 3). This allowed retention of narrative coherence alongside theme development. Cross-case comparison (Ayres et al., 2003) traced thematic convergences and divergences across participants.

#### 3.4.2 Theory-driven thematic and configurational analysis for RQ2

To address RQ2, a theory-driven thematic analysis was conducted using the WCP framework (Braun and Clarke, 2006). The analytic focus shifted from narrative coherence to explanatory abstraction. Because the pre-positioning stage primarily involved biographical background and imagined roles, it was retained narratively but not WCP-coded. WCP coding was applied only to the negotiated and performed stages, where identity shifts were most visible.

Narrative excerpts were coded into WCP dimensions, with directional changes labeled ↑ (increase), ↓ (decrease), or → (stability). Table 2 details the operationalization of WCP, including definitions, indicators, and coding rules for directional shifts (↑ → ↓). Power was examined on two levels: discursive power (Pᵈ) referred to micro-level authority in classroom interaction, institutional discourse and narrative positioning, while structural power (Pˢ) concerned macro-level control through rules, policies and structures. Each coded excerpt was linked to a brief analytic meaning unit that interpreted how WCP changes related to identity movement. Coded data were clustered by positioning stage and synthesized into ternary directional trajectories (e.g., ↑ → ↓), visualizing shifts across the three dimensions.

**Table 2:** Operationalization of the WCP framework.

WCP	Definition (in this study)	Indicators in data	How ↑/↓/→ are judged	Example evidence
Willingness	Motivation or intention to engage in professional actions	Emotional tone (e.g., enthusiasm, burnout); proactive or avoidant behavior	↑: Expressed excitement, reinforced belief ↓: Burnout, disillusionment, detachment →: Stable interest, no major emotional change	↑: *“I’m satisfied with the flexibility; it makes me want to stay.”* (T3) ↓: *“I do not even want to touch thesis supervision anymore.”* (T2)
Capability	Perceived or actual ability to perform institutional or pedagogical tasks	Self-assessment of skills, use of strategies, struggles or improvement in performance	↑: New skills, confidence, successful innovations ↓: Confusion, inefficiency, lack of support →: Adequate but unchanged skill	Teaching →; Research ↓ *“I’m not too worried about teaching; in high school, the pressure and standards were even higher. But thesis supervision feels unfamiliar.”* (T1)
Powerᵈ (Discursive)	Authority enacted through classroom interaction, institutional discourse, and narrative positioning. Reflects micro-level recognition and legitimacy in professional life	Classroom voice, informal feedback, narrative inclusion/exclusion	↑: Gaining recognition or discursive legitimacy (e.g., student affirmation, peer validation) ↓: Being overridden or excluded (e.g., ignored in meetings, feedback dismissed) →: No significant change	↑ *“Some students can become co-creators.”* (T3) ↓ *“I tried to explain. but it did not matter. It’s still your responsibility.”* (T2)
Powerˢ (Structural)	Authority derived from institutional rules, policies, and structures, shaping macro-level access and control	Policy constraints, structural mandates, credential thresholds, or validation requirements	↑: New institutional roles, curricular discretion, or policy support ↓: Denied decision rights, rigid top-down constraints →: Institutional roles unchanged	*“As new teachers without PhDs, we are usually assigned courses only after the professors”* (T2).

**Figure 3:**
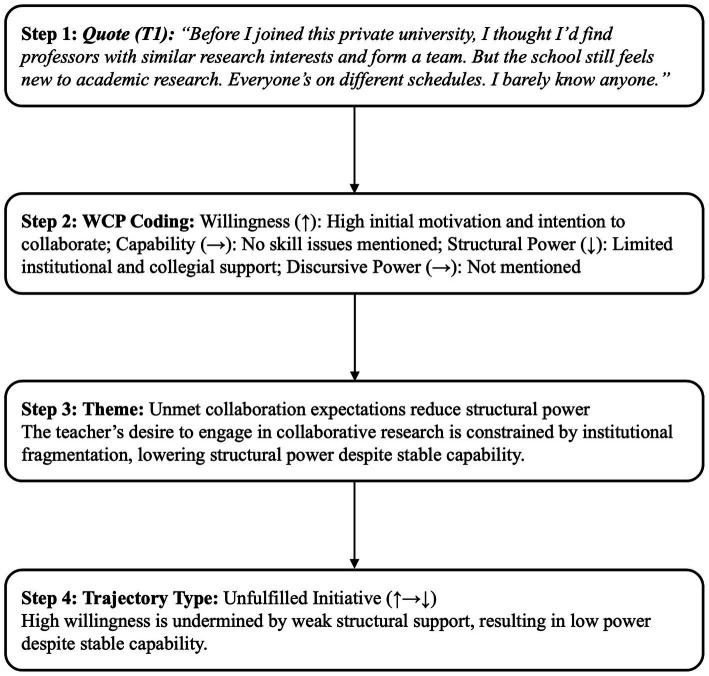
Worked example of WCP coding and trajectory identification.

A configurational analysis was then applied to identify typological patterns across cases. These trajectories are interpretive representations of identity movement rather than statistical measurements. To ensure analytic rigor, only WCP configurations with clear developmental relevance to identity transformation were retained. Two inclusion criteria guided selection: (1) the trajectory must reflect a meaningful shift in willingness, capability, power that altered self- or other-positioning; and (2) the pattern must either recur across two or more narrative episodes or offer strong explanatory insight into how participants navigated institutional or discursive constraints. Configurations failing these thresholds were excluded, including: (1) stable WCP values without identity movement; (2) incremental or ambiguous shifts lacking directional clarity; (3) task-level variations (e.g., emotional fluctuations, procedural remarks) without identity implications; and (4) continuity without transformation despite contextual change. These criteria ensured that the reported four trajectory types in Section 4.2 captured meaningful identity reconfigurations rather than surface-level variation. Figure 3 provides a concrete example illustrating how a narrative excerpt was transformed into WCP codes, themes, and a trajectory pattern. Appendix A presents excluded configurations with justifications; Appendix B provides raw data excerpts and their transformation into final typologies.

The first author independently conducted and continuously refined the coding. To enhance reliability, a second coder, an experienced qualitative researcher and the project supervisor, independently double-coded approximately 10 to 15% of the data. No additional training was required as both were already familiar with narrative inquiry and the WCP framework. Inter-coder agreement was approximately 90%, with fewer than 10% of the segments requiring discussion. Most discrepancies involved subtle distinctions between discursive and structural power, or borderline shifts in willingness. These cases were jointly reviewed and resolved through collaborative interpretation and consensus, consistent with the interpretive nature of the study. To further ensure analytic dependability, an audit log was maintained to document coding decisions, revisions to the codebook, and the rationale behind each interpretive choice. Additionally, analytic memos were kept throughout the process to record emergent insights, reflexive considerations, and the resolution of any discrepancies.

### 3.5 Ethical consideration

This study adhered to ethical principles of respect for the individual, concern for welfare, and justice (Creswell, 2002). Ethical approval for this study was obtained from the institutional ethics committee in China prior to data collection (KY2024-026-01). Written Informed consent was obtained from all participants. The consent form outlined the study’s aims, the voluntary nature of participation, data confidentiality measures, and participants’ right to withdraw at any time without consequences. To maintain confidentiality, we assigned pseudonyms to participants and removed personally identifiable information from the records. The researchers conveyed gratitude for participants’ cooperation by offering small gifts or a nominal financial compensation.

### 3.6 Researchers’ positionality

The intertwining of the positionality of the researcher and the participants informs the telling and retelling of the stories in the query (Clandinin and Connelly, 2000). The first author and participants shared a similar English language teaching background, which, while advantageous, also presents potential biases. This close relationship facilitates a more nuanced understanding of the emotional and institutional pressures within higher education, but it could also lead to assumptions or an overemphasis on teaching details, overlooking broader themes of identity. To mitigate these risks, the analysis employed regular reflective memos, peer feedback, and an analytical triangulation. The corresponding author, an expert in identity research, provided critical feedback throughout the research. This collaborative approach helped ensure the data analysis remained robust, theoretically sound, and unaffected by the first author’s disciplinary perspective.

Classroom and meeting observations were conducted with consent. Researchers did not interfere with participants’ daily teaching schedules or evaluate their teaching practices. Researchers maintained their natural behavior, minimizing the potential for introducing biases into the study. Participants’ WeChat activity was passively observed, limited to occasional “likes” in daily interactions, and analyzed only with explicit participant consent. Given that reflective journals and social media posts may reflect carefully crafted identities, this data was primarily used to provide context rather than dominate narrative interpretation. In addition, the researchers employed techniques such as audit trails, analytical memos, and peer debriefing to ensure the rigor of the analysis and to emphasize reflectiveness in the interpretation process (see Section 3.4). There were no financial or professional conflicts of interest.

## 4 Findings

This section answers the two guiding research questions. In Section 4.1, identity development is recorded through a stage-based narrative analysis spanning three phases: pre-positioning, negotiated positioning, and performed positioning (RQ1). In Section 4.2, the WCP framework is used to assess the interrelation of the institutional mechanisms and discursive dynamics that together provide the structure for four distinct identity trajectories (RQ2).

### 4.1 RQ1: identity across three stages

This subsection addresses RQ1 by describing the three stages of identity development: pre-, negotiated, and performed stages. The key turning points identified through the Critical Incident Technique (CIT) are marked for easy tracking of changes in identity later.

#### 4.1.1 Pre-positioning: contingent entry and gendered framing

These three participants entered EFL teaching at private universities through distinct, nonlinear pathways, shaped by both personal circumstances and the university environment. Here, nonlinear pathways refer to career stories that unfold with breaks, reversals, and emotional or role-related changes, rather than following a smooth or predictable progression.

After completing her doctoral studies in 2023, T1 was ineligible for a position at a public university due to age restrictions: *“Tenure-track positions are generally reserved for candidates under 35” (School recruitment notice, T1).* She also cited health challenges and noted that her supervisor had suggested she consider university teaching. In contrast, T2 described her transition as a more deliberate career choice, driven by dissatisfaction with her corporate job and aligned with her personal development goals: *“I had some training in education, and I felt that being a teacher could help me grow” (Interview, T2).* T3, on the other hand, described a less planned path. After graduating with a master’s degree in 2005, T3 worked at a public university. At first, she mistook it for a high school teacher position. Later, out of her passion for finance, she resigned from the public university. Subsequently, after the 2008 financial crisis, she returned to the education field and has been working in this field ever since *“It wasn’t planned; it just felt natural” (Interview, T3).*

The participants’ accounts also reflected gender discourse, viewing teaching as a stable and family-friendly profession. T2 and T3 referenced their parents’ expectations, with T3 recalling her parents saying, *“Teaching offers more holidays and better stability, especially for women”* (Interview, T3). T1 also mentioned work flexibility and support for children’s education, reflecting the interaction between parenting needs and career decisions. Although this influence was not explicitly reflected in terms of gender.

#### 4.1.2 Negotiated positioning: expectation-reality mismatch, emotional costs, and adaptive reframing

After entering university, all three participants found that there was a discrepancy between their expected roles and the actual environment of the university. T1 described that the way she was guided for her thesis was influenced by her doctoral training and the high academic standards. However, a student complained to the university administration about the way she was guided for thesis *[Complaint Escalation, T1, Negotiated].* In contrast, T2 and T3 were confronted with heavy teaching-related tasks as well as additional work responsibilities, such as handling administrative affairs and supervising students with poor performance. *“There’s just so much meaningless work every day,” (WeChat Post, T2),* while T3 reflected, *“Sometimes I felt like they thought I was the one graduating” (Interview, T3) [Invisible Labor Shock, T2&T3, Negotiated].*

When their professional efforts were not recognized by the university, the participants experienced intense emotional stress. For example, limited research support led T1 to describe herself to an *“academic orphan” [Research Orphaning, T1, Negotiated].* The dissertation led by T2 failed to pass the external review. She discussed this privately and later reflected on it in an interview *[Inspection Failure, T2, Negotiated].* A more detailed description of this turning point can be found in Section 4.2.3. The student evaluations obtained by T3 were lower than expected, which made her start to question her professional competence *[Evaluation Frustration, T3, Negotiated].*

After these events occurred, the three participants adjusted in different ways. T1 lowered her expectations for the supervision of the thesis but continued to provide academic support. T3 incorporated more interactive elements in the classroom activities. In contrast, T2 did not make any significant changes to her teaching or research role during the data collection period.

#### 4.1.3 Performed positioning: roles stabilized, situated specialization, and strategic boundary-setting

After experiencing early identity conflicts, these three participants began to develop a more stable teaching model through situational specialization and strategy adjustment. However, during the data collection, most of T2’s professional narratives remained within the negotiated positioning stage. As a novice teacher without prior experience, T2 was primarily engaged in adjusting to institutional expectations and managing the emotional and administrative demands of her new role. Her performed positioning remained tentative and situational, with no substantial engagement in research.

According to T1, her previous experience in business and secondary education helped her quickly adapt to the teaching requirements of the school. She began to teach business English courses that were in line with the school’s priorities and the constantly changing market demands *[Business English Anchoring, T1, Performed].* T2 devoted herself entirely to the field of teaching and considered herself a *“knowledge deliverer”* (Interview, T2). T3 implemented an e-commerce course following an institutional proposal emphasized real-world business. She reported gaining confidence in designing her own syllabus and expressed satisfaction with the autonomy to adjust content and pace *[E-commerce Course Launch, T3, Performed].*

Participants also enacted boundary-setting strategies. T1 selectively preserved aspects of her academic identity while aligning her teaching with business-oriented institutional goals. T2 expressed detachment from academic pressures, stating *“I stopped wasting energy on inner battles at work” (WeChat, T2).* T3 decided not to pursue a doctorate, explaining: *“I do not need a title. I need a role that fits into my life” (Interview, T3).*

Overall, across the three stages, teachers moved from contingent or gendered entry (pre-positioning), through expectation–reality mismatch and emotional costs (negotiated), to stabilized roles built on specialization and boundary-setting (performed). Identity development was not linear but context-responsive.

### 4.2 RQ2: willingness, capability, power (WCP) trajectories

**Figure 4:**
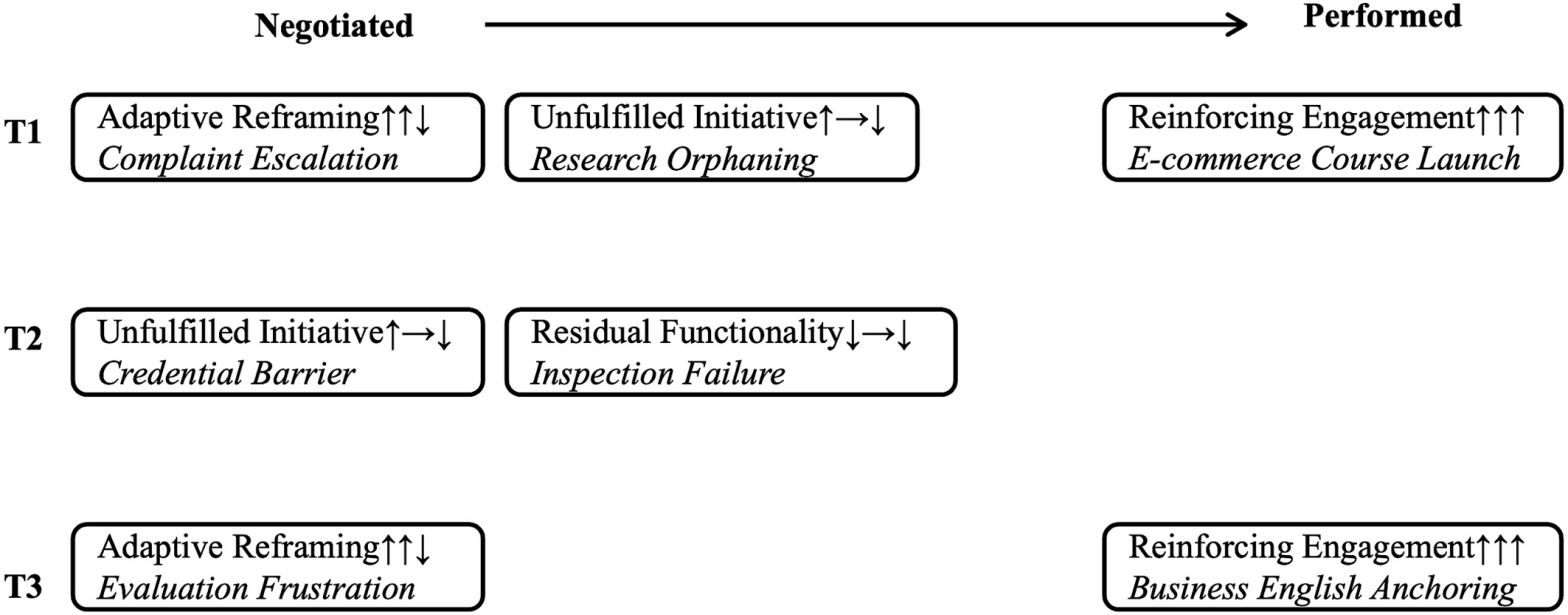
Timeline of teachers’ WCP trajectories across stages.

This subsection answers RQ2 by showing how WCP change over time and form four trajectory types. As defined earlier in Table 2, Pᵈ (discursive power) refers to power in classroom interaction, evaluation and norms, while Pˢ (structural power) refers to power in rules, policies, accountability systems and workload. Together, these two forms of power shape teacher positioning at critical turning points in identity transformation. Table 3 summarizes the WCP shifts across participants and stages, and Figure 4 visualizes these WCP trajectories across the negotiated and performed positioning stages. Although narrative data existed for some stages, such as the performed stage for T2, these were excluded due to a lack of clear or directional WCP shifts.

**Table 3:** Cross-case matrix of WCP shifts and identity trajectories by stages.

Teacher	Stage	Turning points	W	C	P^d^	P^s^	Trajectory	Pattern quotes
T1	Negotiated	Research collaboration unmet → individualized responsibility	↑	→	↓	↓	Unfulfilled Initiative (↑ → ↓)	*“I barely know anyone”*
T1	Negotiated	Evaluation pressure → authority reframed	↑	↑	↓	NA	Adaptive Reframing (↑↑↓)	*“You cannot expect to have absolute authority”*
T1	Performed	Curriculum redesign → student-centered tasks	↑	↑	↑	NA	Reinforcing Engagement (↑↑↑)	*“Students prefer real-world tasks”*
T2	Negotiated	Thesis inspection failure → loss of voice	↓	→	↓	↓	Residual Functionality (↓ → ↓)	*“I was so angry I even wanted to resign”*
T2	Negotiated	Credential barrier → limited research role	↑	→	↓	↓	Unfulfilled Initiative (↑ → ↓)	*“Only PhDs are expected to research”*
T3	Negotiated	Negative student evaluation → pedagogical shift	↑	↑	↓	NA	Adaptive Reframing (↑↑↓)	*“I’m not Chinese yuan”*
T3	Performed	E-commerce course launch → inquiry-based facilitation	↑	↑	↑	↑	Reinforcing Engagement (↑↑↑)	*“Students became co-creators”*

NA = No rule or policy changed in this episode; in such cases, trajectories were inferred based on discursive evidence.

#### 4.2.1 Unfulfilled initiative: willingness increased, capability unchanged, power decreased (↑ → ↓)

This trajectory type reflects cases where teachers maintained high willingness and stable capability but experienced a decline in both structural (e.g., limited access to research resources, credential-driven role allocation) and discursive (e.g., individualization of professional responsibility, marginalization from institutional research discourse) power. These tensions are illustrated in T1’s experience of constrained collaboration and T2’s negotiation of credential-based limitations.

*T1 in Figure* *4**: Unfulfilled Initiative after Research Orphaning [Negotiated Stage].*

T1 described her expectation for research collaboration upon entering the institution:

*Interview (T1, negotiated stage): “Before I joined this private university, I thought I’d find professors with similar research interests and form a team. But the school still feels new to academic research. Everyone’s on different schedules. I barely know anyone.”*

The statement depicts a positive attitude towards collaborative research participation (W↑), however, statements like *“new to academic research”* also shows a lack of relevant underlying institutional mechanisms (Pˢ↓), such as lack of research network or collegiality. This lack of institutional support is further confirmed by formal policies:

*Document excerpt (T1, negotiated stage): 2025 Research Evaluation Guidelines “Failure to meet minimum publication requirements may have an impact on annual assessment performance and promotion eligibility.”*

Rather than supporting research development, this policy has increased pressure through a results-oriented evaluation approach. Meanwhile, T1’s discursive power (Pᵈ↓) has been weakened by the institutional discourse which focuses on personal achievements.

*Fieldnotes (T1, negotiated stage): During department meetings, it was emphasized that “You’re expected to manage your own output.”*

This narrative positions T1 as a self-managing performer rather than a collaborator. Despite her stable research capabilities (C→), structural and discursive disempowerment constrains her professional identity, which results in a WCP trajectory (↑ → ↓) that aligns with the Unfulfilled Initiative path.

*T2 in Figure* *4**: Unfulfilled Initiative after Credential Barrier [Negotiated Stage].*

T2, a young teaching assistant without a doctorate, encountered structural constraints imposed by the academic hierarchy.

*Interview (T2, negotiated stage): “We’re usually assigned courses only after the professors. If the school officially stops hiring master’s degrees, I’ll probably have to pursue a part-time PhD just to keep up.”*

The phrase *“assigned courses only after the professors”* illustrates an allocation mechanism that privileges academic credentials over teaching performance (Pˢ↓). Though T2 showed interest in pursuing a doctoral degree (W↑), the phrase “just to keep up” conveys her need to fit in rather than take initiative or be creative. Although her abilities are not diminished, they are not acknowledged (C→).

In terms of research-related expectations, her positionality was also strictly circumscribed by the institutional narrative:

*Interview (T2, negotiated stage): “The institution only expects PhD holders to do research; we primarily teach.”*

Using *“PhD holders”* as a proxy for who is responsible for research signifies the use of academic credentials as a policy approach that structurally limits participation (Pˢ↓), combining a discursive strategy that effectively ignores non-PhD educators from the research community (Pᵈ↓). T2’s identity transitioned into one of compliance as the structural and discursive frameworks left little space for her professional growth. In T2’s case, as with T1, her profile (W↑, C→, Pˢ↓, Pᵈ↓) demonstrates the pattern of Unfulfilled Initiative (↑ → ↓).

In all these instances, teachers’ autonomy and epistemic voice were diminished by institutional regulations and individualizing discourse. This is what Skog and Andersson (2015) call institutional scripting, which implicitly controls who may lead, innovate, or speak even when there is professional competence present.

#### 4.2.2 Adaptive reframing: willingness increased, capability increased, power decreased (↑↑↓)

This part analyses how teachers with motivation and capability adapt their professional identities in contexts where discursive authority is limited despite stable structural conditions. This balances the need to conform to the institutional expectations while still retaining the central value. T1 and T3 are exemplary in that regard: T1 shows flexibility with students during the thesis supervision process and T3 navigates the evaluative pressures of teaching.

*T1 in Figure* *4**: Adaptive Reframing after Complaint Escalation [Negotiated Stage].*

As mentioned in 4.1.2, the student complaint to the department that challenged the high standards of T1’s thesis supervision. This marked the beginning of a shift in how her authority was perceived within the institution.

*Interview, (T1, negotiated stage): “I gradually realized that teaching here is more about being able to communicate with students. You cannot expect to have absolute authority, so I adjusted my thesis academic standards to try to prevent such complaints from happening again.”*

In this statement, T1 actively engaged in reflection and adjustment (W↑). However, her academic expertise, once grounded in disciplinary authority, gradually lost its discursive legitimacy within the student-centered evaluative logic of this private university, where satisfying student expectations increasingly replaced traditional academic criteria (Pᵈ↓). In response, T1 redefined her competence by shifting from a disciplinary expert to a relational communicator (C↑). This redefinition did not indicate a rejection of expertise, but rather a realignment with what now counted as expertise in her context. Her feedback to students reflects this new orientation:

*Thesis Feedback (T1, negotiated stage): “It’s okay if it’s not perfect now; we’ll refine and improve it step by step.”*

Despite her personal adjustments, the broader institutional structures remained unchanged (Pˢ→), as student satisfaction has now become a quasi-management mechanism in Chinese private universities, limiting her ability to reassert professional standards through institutional means. T1’s shift signaled a discursive realignment in which evaluative authority was redefined by responsiveness rather than epistemic standards. Her reorientation toward a more service-oriented facilitator role exemplifies identity negotiation within a managerialized system. Her trajectory, therefore, mirrors the ↑↑↓ pattern, an example of Adaptive Reframing (discursive pressure).

*T3 in Figure* *4**: Adaptive Reframing after Evaluation Frustration [Negotiated Stage].*

As described in Section 4.1.2, T3’s frustration with evaluation prompted her to shift her position in the classroom as she redefined her teaching role to better align with student expectations.

*Interview, (T3, negotiated stage): “I spent a lot of time preparing and explaining the material in detail, but my student ratings were much lower than those of teachers who mostly played videos. It made me question myself and felt really down. I tried to comfort myself. I’m not Chinese yuan; not everyone will like me. So, I focused more on engaging with my students.”*

*Observation (T3, negotiated stage): “She adapted her delivery using humor, varied pacing, and short videos, which made the lessons more accessible while maintaining academic rigor.”*

*Reflective Writings (T3, negotiated stage): I don’t want to become a clown, but I have to survive the evaluations.*

T3 experienced a decline in discursive power (Pᵈ↓) when her commitment to academic rigor did not translate into positive teaching evaluations. Her subject knowledge and preparation no longer carried institutional weight in the eyes of student evaluators. T3’s reflective writings also captures the tension between preserving professional integrity and adapting to an evaluative regime driven by student popularity.

**Figure 5:**
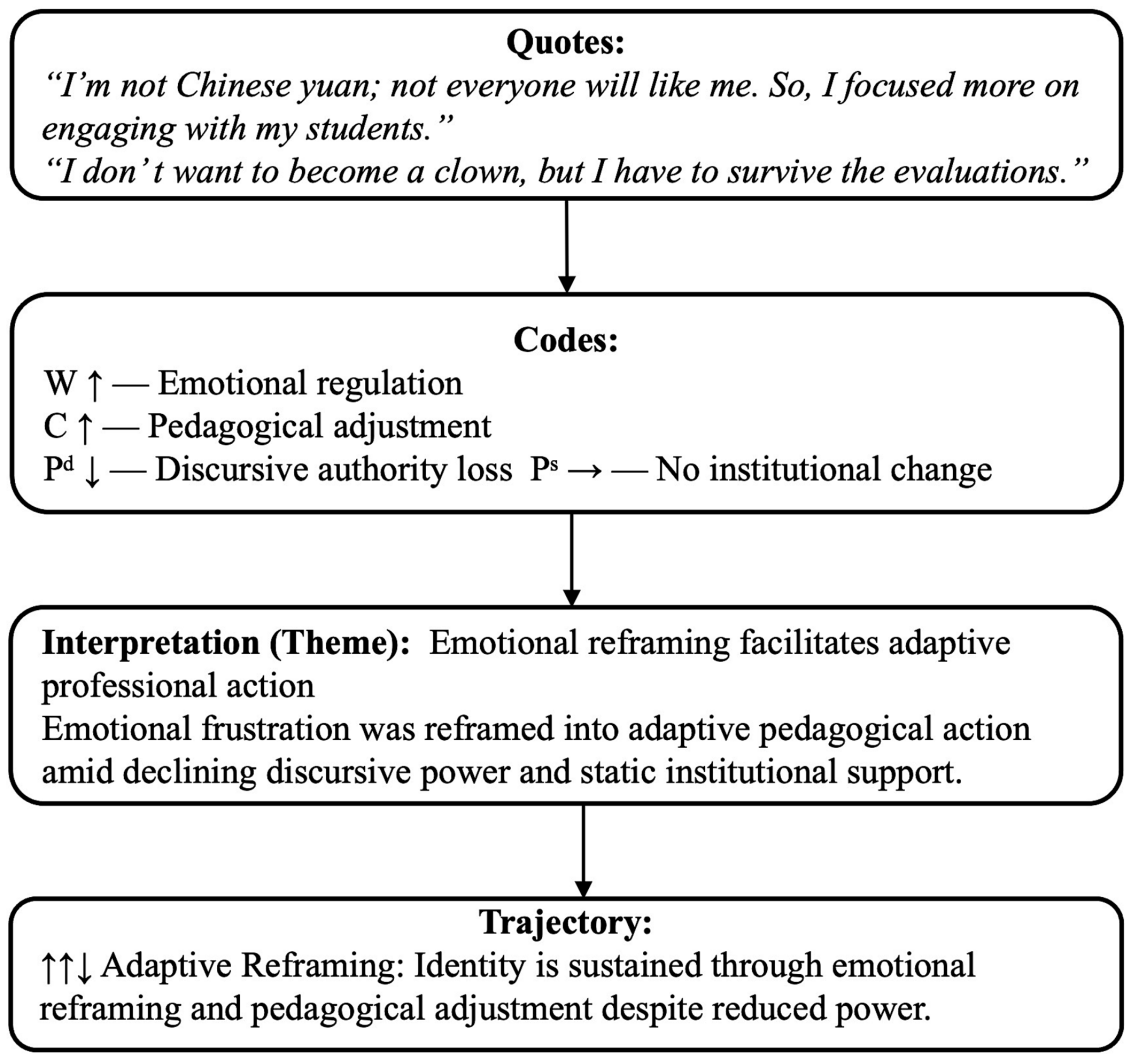
Worked example of adaptive reframing (↑↑↓) from T3 in negotiated stage.

In response, T3’s emotional regulatory response (*“comforting myself”*) and sustained professional effort demonstrated a high level of willingness (W↑). She enhanced her teaching competence (C↑) by adopting a more flexible, student-centered approach while maintaining academic standards. These adjustments reflect a reconfiguration of her professional identity, from a transmitter of knowledge to a facilitator of relationships. This shift stemmed from classroom adaptation rather than structural change (Pˢ→). Her micro-positional adjustments reflect an *Adaptive Refram*ing path (↑↑↓), characterized by emotional adaptation and localized classroom strategies. This process is visually illustrated in Figure 5.

Together, this path illustrates teachers who actively recalibrated their supervisory roles, pedagogical practices, and emotions to sustain professional legitimacy under discursive constraint. While negotiating with students and responding to their needs is not inherently disempowering, for T1 and T3, such negotiations unfolded within an evaluative system where student satisfaction had overtaken academic authority as the dominant performance metric. Unlike those in the Unfulfilled Initiative path, they employed localized strategies to uphold professional values despite limited power. In contrast to Wu’s (2022) teacher, whose transformation followed structural mobility through PhD pursuit, T1 and T3 repositioned themselves through affective and classroom-level adjustments.

#### 4.2.3 Residual functionality: willingness decreased, capability unchanged, power decreased (↓ → ↓)

Residual functionality refers to teachers continued professional performance under emotional withdrawal and diminished authority. It reflects a survival-oriented professionalism, where compliance replaces commitment. In T2’ s case, both discursive power (e.g., voice in supervision and departmental discourse) and structural power (e.g., institutional accountability systems) declined simultaneously.

*T2 in Figure* *4**: Residual Functionality after Inspection Failure [Negotiated Stage].*

As noted in Section 4.1.2, T2 retrospectively described the thesis review incidence as a *“professional Waterloo.”* Rather than asserting resistance, she articulated internalized disorientation:

*Private journal, (T2, negotiated stage): I was so angry that I even wanted to resign.”*

*Interview, (T2, negotiated stage): “I truly felt I had guided the student with care, but the result was far from what I had hoped for. It shook my confidence and made me question my academic ability.”*

Expressions like *“angry” “resign”* reveal a collapse of intrinsic motivation (W↓). Her emphasis on *“guided the student with care”* suggests she still considers herself competent in thesis supervision (C→). However, the institution’s results-based evaluation system fails to recognize this effort, limiting T2’s opportunities to justify the situation and weakening her discursive voice (Pᵈ↓). She reflects on beginning to *“question [her] academic ability,”* suggesting that the academic self-concept once built on institutional trust has begun to erode.

This loss of discursive power also emerged in informal interaction:

*WeChat Chatlog (T2, negotiated stage): “Half my students are doing internships. Some even say ‘I’m too busy with work to focus on the thesis.’”*

*Interview, (T2, negotiated stage): “I tried to explain how the student had repeatedly ignored my advice, but it did not matter. The attitude was: it’s still your responsibility.”*

T2’s narrative reflects a disconnect between the institutional accountability framework and students’ perceived academic disengagement. The phrase *“too busy with work to focus”* normalizes students’ disengagement and undermines the legitimacy of her supervising efforts. T2 attempted to explain students’ poor performance, but these attempts were rejected, limiting the space for discussion necessary for constructing professional meaning. These denials reinforced a deficit identity and negated her efforts to reposition herself through rational explanation (Pᵈ↓).

Structurally, she described a one-sided accountability system:

*Interview, (T2, negotiated stage): “All the pressure is on us, endless meetings for teachers, none for students. If anything goes wrong, only the thesis advisor is penalized; the students, defense panel and review faculty are unaffected.”*

**Figure 6:**
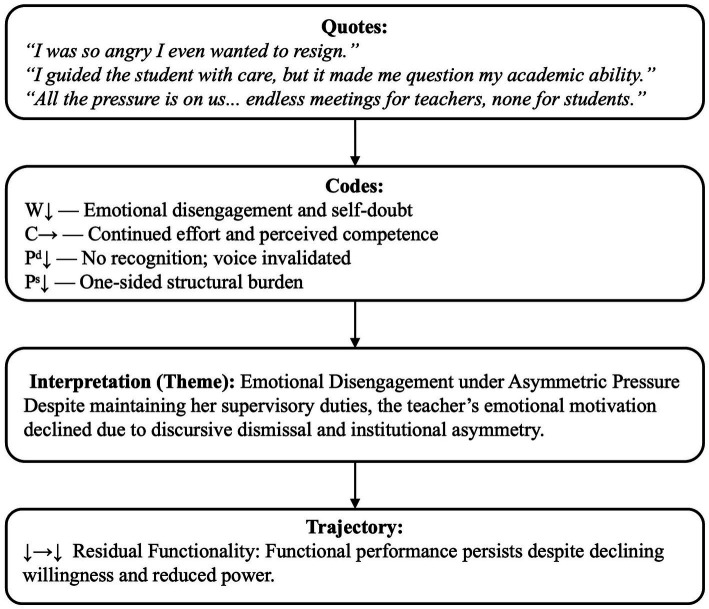
Worked example of residual functionality (↓ → ↓) from T2 in negotiated stage.

T2’s comments reveal an asymmetrical accountability structure, in which faculty are monitored while others are unfettered. The contrast between *“endless meetings for teachers”* and *“none for students”* illustrates an unequal system of institutional burden (Pˢ↓). Although she continued to supervise (C→), her emotional investment decreased (W↓). Figure 6 presents an example from T2’s case to illustrate how emotional disengagement and structural burden form a trajectory of *Residual Functionality* (↓ → ↓).

This case illustrates teachers who continued to fulfill their responsibilities, albeit with a certain emotional detachment and loss of authority. In contrast to those on the Adaptive Reframing path, who purposefully reframed their practices in meaningful ways, these teachers simply complied. They lacked the agency to reframe their practices, bound as they were by discursive silencing and structural constraints. They performed the tasks, but recognition, voice, and revitalization were absent. Her case elaborates on Shi’s (2022) account of institutional misalignment by demonstrating how sustained discursive silencing creates an environment conducive to withdrawal as opposed to recalibration.

#### 4.2.4 Reinforcing engagement: willingness increased, capability increased, power increased (↑↑↑)

Reinforcing engagement is the intertwined development of willingness, capacity, and discursive authority, maintained through classroom identity repositioning. While some initial opportunities may come from structural power, it is through discursive acts, like inquiry-based facilitation or framing as a practitioner, that teachers use power and transform their roles. In this study, T3 and T1 serve as exemplars of two varieties of this engagement, the former shaped by experiential pedagogy in sync with institutional innovation (T3), and the latter framed by professional expertise and student responsiveness (T1).

*T3 in Figure* *4**: Reinforcing Engagement after E-commerce Course Launch [Performed Stage].*

Building on the structural support (Pˢ↑) mentioned in Section 4.1.3, T3 seized this opportunity to leverage an inquiry-based teaching approach that prioritized student participation.

*Observation (T3, performed stage): In class, she facilitated inquiry, encouraged experimentation with marketing tools, and explicitly praised those who expressed independent insights and engaged in academic exploration.*

Her willingness to relinquish control and experiment with new strategies demonstrated her openness to repositioning her identity (W↑). Her recognition of students’ active contributions was later elaborated upon in her reflections:

*Interview, (T3, performed stage): “When a student used a platform I had not taught, I realized he wasn’t just following my steps but was exploring and self-learning. That’s when I saw I’m not the only knowledge provider; some students can become co-creators.”*

Repositioning students as *“co-creators”* signaled a shift in classroom authority and affirmed T3’s evolving role as a facilitator. Her discursive power (Pᵈ↑) is reflected in how she embraces the shared knowledge space, while her growing capacity (C↑) enables her to guide student-centered exploration and manage dynamic inquiry-based learning. The institutional empowerment, the adjustment of teaching strategies, and the reshaping of her identity collectively embody the path of *Reinforcing Engagement* (↑↑↑).

*T1 in Figure* *4**: Reinforcing Engagement after Business English Anchoring [Performed Stage].*

Despite lacking sufficient institutional support, T1 enacted discursive power by independently developing her curriculum (Pᵈ↑), leveraging her business background to enhance the relevance of her courses and strengthen connections with students.

*Interview, (T1, performed stage): “I can bring in real logistics or trade knowledge. Students seem to prefer it when I include interesting or practical content drawn from real business experiences.”*

The willingness which was indicated through her student response (W↑), and her teaching capability as shown through the designed applied tasks of trade documents and transactional emails (C↑). The practical educator position and the epistemic legitimacy gained through lived business experience (Pᵈ↑).

These cases highlight Reinforcing Engagement as a trajectory of identity development susteined by the consonance of the teachers’ beliefs and the classroom realities. In both cases, unlike the more subtle institutional support (more pronounced in T3), both teachers exercised agency in their pedagogy and their role-centric choices. Reinforcing Engagement, unlike Adaptive Reframing, which arose under discursive pressure, pertains to identity development of a broad spectrum that encompasses recognition, autonomy, and meaning co-creation. This builds on previous research (e.g., Kayi-Aydar, 2018; Sun et al., 2022) that claims the teacher’s identity encompasses not only the realities of constraint, but the realities of opportunity as well.

In summary, teachers’ identity transitions occurred along four trajectories due to different combinations of WCP. The trajectory of Unfulfilled Initiative (↑ → ↓) emerged when strong motivation was counters by a decline in structural or discursive power. Adaptive Reframing (↑↑↓) described cases where, due to a decline of discursive power, they adjusted their practices to remain engaged. Residual Functionality (↓ → ↓) reflected continued task performance but in an emotionally drained state that reflected a loss of power. Reinforcing Engagement (↑↑↑) represented contexts when motivation, capability, and the conditions for enabling all came together. These four patterns indicate that WCP is not a static property, but inevitably a dynamic function of the prevailing institutional and discursive contexts. For instance, T1 moved from Unfulfilled Initiative to Reinforcing Engagement.

## 5 Discussion

This research focuses on the formation, negotiation, and enactment of English teacher identity, as well as the ways in which WCP dynamically shape the information of positioning trajectories under structural and discursive constraints in the context of Chinese private tertiary institutions. This study contributes to psychological understandings of teacher identity by showing how emotional disruption, discursive recognition, and fluctuating agency resources jointly shape identity regulation in constrained contexts.

The findings regarding RQ1 complicate the existence of stage-based models of individual identity development. At the pre-positioning stage, the findings challenge the assumption that identity begins with aspirational entry and reflective practice (Tsui, 2007). For many participants, however, aspirational identities were absent at the point of entry. Their initial positioning was shaped by factors such as life trajectory dislocation (e.g., disillusionment with the organization at T2), structural exclusion (e.g., T1 age-based recruitment restrictions), and gendered rationalizations (e.g., the idealized notion that teaching is a stable position for women). These forces, which work in conjunction with the labor market (Mockler, 2011) and cultural narratives (Gaskell and Mullen, 2006), give rise to passive identity formation. Lastly, these findings imply that onboarding processes should involve more than merely technical orientation; rather, they should also involve the scaffolding of identity transformation that recognizes the wide range of entry points and ambivalence.

At the negotiated positioning stage, the processes of identity negotiation are not simply a process of dialogue and cognitive shift (Davies and Harré, 1999). In the private-university context of this study, identity negotiation frequently unfolded in response to unresolved emotional disruption, dissonance, and the need for emotional regulation. Extending Xing et al. (2025), this study analyzed teachers’ emotions within the institutional framework, emphasizing that emotions are not individual issues but are influenced by institutional arrangements and are managed in a specific manner. For instance, student-centered powers (e.g., evaluation) can increase the vulnerability of teachers. These results suggest the need for institutional support which goes beyond material support, to include emotional support, such as peer mentoring, reflective counselling, and structured system space discourse scaffolding.

In the performed positioned stage, this study adds nuance to the identity stability assertion of Wu (2022) and Zhang (2025) claiming that it hinges on mobility, academic credentials, or institutional recognition. Instead, it suggests that stability is often achieved through adaptive repositioning in response to institutional constraints (Arvaja, 2016). Teachers sustain their identities by selectively complying with organizational expectations and negotiating professional meaning around either practical teaching goals or personal values. Such strategies reflect the negotiability of discursive borders and the reframing of identity as a nonlinear path; that is, not a predictable ascent toward recognition, but a process shaped by detours, compromises, and emotional recalibration (Beijaard et al., 2004). Therefore, it is imperative that organizations move beyond a metrics-dominated paradigm and focus on the emotional burdens experienced in constrained settings.

As an answer to RQ2, the study revealed four different identity trajectories linked to the shifting WCP configurations at the various stages of positioning. The Unfulfilled Initiative trajectory (↑ → ↓) redefines agentive models of teacher identity (Weng, 2023) by demonstrating that agency, defined here as sustained willingness and stable capability, may remain unrealized when teachers are discursively unrecognized or structurally excluded. While Sun et al. (2022) underscored the importance of contextual affordances such as institutional support and collaboration, our findings highlight a critical distinction: contextual affordances alone are insufficient when teachers are discursively unrecognized. This pattern was evidenced in both T1’s constrained participation in research-related discourse and T2’s credential-based exclusion from academic decision-making. In such cases, these teachers are expected to manage their work alone rather than be included as knowledge contributors, which limits their access to collaboration, academic decision-making, and recognition. To conceptualize this mechanism, we introduce the notion of *discursively orphaned identity.* This refers to cases where teachers hold formal institutional roles yet experience silencing, non-recognition, or restricted discursive space within their narrated experiences, as shown by strong willingness and stable capability that nevertheless fail to translate into recognized participation. This aligns with Skog and Andersson’s (2015) view of institutional scripting, wherein implicit norms regulate who is permitted to lead, innovate, or speak, regardless of capability. Drawing on Foucauldian perspectives (Foucault, 1980), we view power and knowledge as co-constituted. From this perspective, teachers may be institutionally visible yet epistemically marginalized, formally recognized in role but denied the discursive power needed to claim legitimate professional positions. To solve this problem, more inclusive career development paths are needed to recognize various forms of participation and professional knowledge.

The Adaptive Reframing trajectory (↑↑↓) considers the strategic, subtle shifts teachers are willing and able to respond to discursive disempowerment make to their practices. Extending Huang and Wang’ s (2024) typology of identity responses, we propose a fourth mode: adaptive accommodation, whereby teachers respond to symbolic marginalization not with overt opposition, but through emotional labor and relational positioning that maintain institutional viability. Indeed, in private universities, emotional acceptability prevails over the demands in the academy, and teachers enact affability in lieu of epistemic authority. They engage in affective and discursive labor to maintain a professional identity instead of pursuing public academic recognition (Wu, 2022). Theoretically, this trajectory refines positioning theory by emphasizing non-oppositional identity negotiation in constrained environments, and expands the WCP framework by showing how increased willingness and capability (W↑, C↑) can offset declining discursive power (P↓). It offers a third space between compliance and resistance, where professional value is re-authored through affective positioning.

Residual Functionality (↓ → ↓) reflects identity stagnation among teachers wherein they continue institutional duties, but neither the opportunity, nor the discursive space, to explain their actions permits for symbolic erosion. When compared to role misalignments (Shi, 2022), this pattern suggests that the primary cause of teacher detachment is the inability to articulate professional meaning, rather than simply the structural overload. In such contexts, responsibility is allocated without recognition, and accountability operates without dialogue. To conceptualize this dynamic, we propose the term *performed stagnation*, referring to identities that are enacted through outward compliance but remain unsupported by discursive affirmation. This mechanism was reflected most clearly in T2’s trajectory following the inspection failure, where emotional withdrawal coexisted with sustained task performance amid declining structural and discursive power. While Weiß et al. (2023) indicate that professional identification grows in autonomy-supportive contexts, our data suggest that sustained commitment alone may be insufficient when teachers lack the discursive space to justify their work. In private universities, the one-way accountability structure suppresses professional voices and causes moral fatigue, as teachers are only required to be responsible for the results but are not invited to participate in the creation of meaning. Here, silence rather than open conflict becomes the mechanism that erases the professional presence. Drawing on Foucauldian analyses of discourse and power (Foucault, 1970, 1980), we interpret silence not merely as personal withdrawal, but as a discursive effect of institutional power. It is a mechanism of institutional power that determines whose voices are legitimized and whose knowledge remains unacknowledged. Restoring the agency of discourse requires institutional embedding in narrative assessment forms and the cultivation of dialogic accountability mechanisms, which should be able to recognize that the voices of teachers are crucial for professional identity.

The trajectory of Reinforcing Engagement (↑↑↑) indicates that the growth of identity can be achieved through the alignment of various forms of values and practices. Rather than relying solely on institutional mandates, this trajectory demonstrates how teachers exercise agentive repositioning to construct legitimacy in loosely regulated spaces. By drawing on teaching expertise and affective commitment, they shape the terms of their engagement, not merely respond to them. These cases expand the positioning theory, enabling it to go beyond passive adaptation, demonstrating how teachers can actively shape discourse and construct legitimacy in loosely defined or less supervised spaces. In this sense, enhanced participation is not only dependent on structural availability. It may also stem from active repositioning, that is, using teaching expertise to maintain the right to speak. This requires institutions to create flexible environments to promote teaching innovation, affirm emotional pressure, and support the joint construction of meaning by teachers and students.

These findings offer several action-oriented implications, particularly for administrators of private universities. For example, supporting faculty development requires moving beyond procedural induction training and shifting towards a focus on diverse induction guidance. Alternatively, private universities should foster an emotionally and discursively supportive environment and recognize diverse forms of contribution, such as pedagogical innovation, within career development pathways.

Together, these findings collectively expand upon existing understandings of language teacher identity, demonstrating that changes in the identity of private university faculty are deeply influenced by emotional stress, discursive vulnerability, and the fluctuating configurations of WCP. In this context, teacher identity evolves through adaptive and often strategic repositioning to cope with constrained evaluation systems, limited legitimacy pathways, and unequal opportunities. These insights highlight the need to understand language teacher identity not only through its fluid but also by the institutional, emotional, and power-loaded conditions unique to private higher education that shape it.

## 6 Conclusion and limitations

This study examined how English teachers in a Chinese private university negotiated, sustained, or transformed their identities under shifting configurations of willingness, capability, power (WCP). By analyzing narratives across pre-positioning, negotiated positioning, and performed positioning stages, the study challenges linear models of identity development and highlights the role of emotional disruption, discursive vulnerability, and institutional constraint in shaping teacher identity. By identifying four pathways, Unfulfilled Initiative, Adaptive Reframing, Residual Functionality, and Reinforcing Engagement, this research develops positioning theory to incorporate a temporal and power-sensitive approach to examine the flexibility and fragility of teacher identity in context of under-resourced privatized universities. The findings demonstrate that identity change is fundamentally a psychological phenomenon of managing emotions and regulating one’s thought processes, and an enactment of agency.

Several limitations should be noted. First, the study was conducted at a single private university in southeastern China. While this enabled deep contextual engagement, it may limit the generalizability of findings. Future research should include diverse institutional types and regions to enhance variation. Second, all participants were female. Although this is influenced by the gender composition of the department, it also limits our understanding of how gender expectations are intertwined with identity positioning. Third, the small sample size may introduce structural bias. However, this aligns with the characteristic of narrative inquiry that emphasizes depth rather than breadth. Fourth, this study employed self-reported narratives collected through interviews, reflective diaries, and WeChat communications. Such data are inherently selective and may introduce recall or impression-management bias. To reduce these risks, multiple narrative sources were triangulated, critical incidents were checked across data points, and observations were used to provide contextual grounding. Finally, the dual-layered analytic design, combining stage-based narrative analysis with WCP configurational analysis, may appear complex given the sample size. While this enhanced interpretive depth by integrating narrative flow with theoretical abstraction, it also risks over-interpretation. Future studies with larger and more diverse samples are needed to test the robustness and transferability of the four identity trajectories identified.

## Data Availability

The raw data supporting the conclusions of this article will be made available by the authors, without undue reservation.
