# Supplementary material for: Positioning, power, and identity transformation among English teachers in Chinese private universities: a narrative inquiry
Source: Front Psychol. 2026 Jan 12;16:1729332. doi: 10.3389/fpsyg.2025.1729332 (PMC12833011; doi:10.3389/fpsyg.2025.1729332)
Supplement: Supplementary file 1 [file Supplementary_file_1.docx]

Supplementary Material

**Appendix**

**Appendix A Illustrative Examples of Excluded WCP Configurations and Thresholds for Inclusion**

This Appendix A presents narrative excerpts that were excluded from typology construction based on the analytic thresholds outlined in Section 3.3. Each example failed to demonstrate sufficient identity transformation or lacked explanatory strength, as defined by the inclusion criteria. Codes and directional WCP patterns are included, along with justifications for exclusion.

| Quote | Stage | WCP Pattern | Rationale for Exclusion |
| --- | --- | --- | --- |
| *“I often apply for projects and work closely with the dean. For national-level MOE projects, the university provides expert reviews and multiple revisions as part of its standard process.” (T1)* | Negotiated Stage | **Willingness (↑):** Strong motivation to engage in research and institutional procedures.  **Capability (→ ):** Active involvement, but no sign of growth or identity shift.  **Power** ˢ**(↑):** Gains structural support through leadership and expert feedback. | Although this reflects high willingness and structural support (power↑), there is no evidence of capability growth or identity repositioning. The teacher continues in an administrative-research alignment, but without developmental shifts relevant to identity transformation. |
| *“I used to think students were self-disciplined, but some won’ t submit assignments without repeated reminders even before the final exam.”(T2)* | Negotiated Stage | **Willingness (→):** Continued effort to manage students despite unmet expectations.  **Capability (→):** No evident development in teaching skills. **Power** ᵈ **(↓):** Repeated reminders were ineffective, indicating weak micro-level control. | This configuration shows stable willingness and capability, with limited classroom authority and no evidence of professional growth or identity shift. It was excluded due to limited theoretical relevance to identity transformation. |
| *“Teachers should first have a positive attitude and complete the tasks assigned by the school. However, they also need to balance work and family, and keep learning to avoid falling behind.” (T2)* | Performed Stage | **Willingness (→):**Sustained engagement, but primarily framed as responsibility rather than passion.  **Capability (↑):**Awareness of continuous learning.  **Power (→ ):** No evidence of institutional recognition or greater voice; no change in workload or institutional affordances. | This episode reflects emerging self-regulation, suggesting partial recovery from earlier disengagement. However, the change is incremental and does not constitute a distinct WCP trajectory. It was excluded from the typology but demonstrates how teachers may reframe commitment without structural or discursive empowerment. |

**Appendix B Illustrative WCP Coding: From Narrative Episodes to Identity Trajectories**

This Appendix B provides representative examples of how narrative excerpts were coded into WCP dimensions and assigned to one of the four typological trajectories. Each row includes the participant, stage, coded dimensions, and the resulting WCP configuration.

| Participant | Narrative Episode | Stage Label | W | C | Pˢ | Pᵈ | Theme | WCP Trajectory |
| --- | --- | --- | --- | --- | --- | --- | --- | --- |
| T2 | *“All the pressure is on us, endless meetings for teachers, none for students. Only the supervisor is penalized; the students, defense and review faculty are unaffected. Honestly, I don’t even want to touch thesis supervision anymore.”* | Stage 2 (Negotiated Stage) | ↓  Emotional exhaustion | → Stagnant capability not due to skill deficit | ↓  Asymmetric responsibility; imposed accountability. | → | Withdrawing from undervalued academic labor | ↓→↓ Residual Functionality |
| T3 | *The institution hopes that I can lead students to sell products and engage in real business.* | Stage 3 (Performed Stage) | ↑ Professional openness to innovative pedagogical tasks | ↑ Practice-based instructional competence | ↑ Increased institutional trust and assigned responsibility | → | Aligning with institutional goals through practice-based teaching | ↑↑↑ Reinforcing Engagement |
